# Embryonic/fetal mortality and intrauterine growth restriction is not exclusive to the CBA/J sub-strain in the CBA × DBA model

**DOI:** 10.1038/srep35138

**Published:** 2016-10-21

**Authors:** Kelly J. McKelvey, Vanessa M. Yenson, Anthony W. Ashton, Jonathan M. Morris, Sharon A. McCracken

**Affiliations:** 1 Division of Perinatal Medicine, Kolling Institute, Northern Sydney Local Health District, St Leonards, 2065 NSW, Australia; 2 Sydney Medical School Northern, University of Sydney, St Leonards, 2065 NSW, Australia; 3 Department of Obstetrics and Gynaecology, Royal North Shore Hospital, Northern Sydney Local Health District, St Leonards, 2065 NSW, Australia

## Abstract

Inbred strains of mice are powerful models for understanding human pregnancy complications. For example, the exclusive mating of CBA/J females to DBA/2J males increases fetal resorption to 20–35% with an associated decline in placentation and maintenance of maternal Th1 immunity. More recently other complications of pregnancy, IUGR and preeclampsia, have been reported in this model. The aim of this study was to qualify whether the CBA/CaH substrain female can substitute for CBA/J to evoke a phenotype of embryonic/fetal mortality and IUGR. (CBA/CaH × DBA/2J) F1 had significantly higher embryonic/fetal mortality mortality (p = 0.0063), smaller fetuses (p < 0.0001), and greater prevalence of IUGR (<10^th^ percentile; 47% vs 10%) than (CBA/CaH × Balb/c) F1. Placentae from IUGR fetuses from all mating groups were significantly smaller (p < 0.0001) with evidence of thrombosis and fibrosis when compared to normal-weight fetuses ( > 10^th^ percentile). In addition, placentae of “normal-weight” (CBA/CaH × DBA/2J) F1 were significantly smaller (p < 0.0006) with a greater proportion of labyrinth (p = 0.0128) and an 11-fold increase in F4/80 + macrophage infiltration (p < 0.0001) when compared to placentae of (CBA/CaH × Balb/c) F1. In conclusion, the embryonic/fetal mortality and IUGR phenotype is not exclusive to CBA/J female mouse, and CBA/CaH females can be substituted to provide a model for the assessment of novel therapeutics.

In the last century there has been a vast improvement in overall perinatal health. However, high rates of perinatal mortality and morbidity persist in spite of advances in medical technologies and management, such as routine use of ultrasound to monitor fetuses during pregnancy. An important cause of perinatal mortality, secondary only to preterm birth, is the failure of the fetus to reach its growth potential *in utero*, termed intrauterine growth restriction (IUGR); defined by the World Health Organisation as an estimated fetal weight below the 10^th^ percentile of the recommended gender-specific birth weight for gestational age reference curve^1^. Worldwide, IUGR affects 7–15% of pregnancies^2^,^3^ and has an 8–10-fold greater risk of perinatal mortality when compared to pregnancies with a normal-weight fetus^4^.

Mouse models are a powerful tool for understanding pregnancy outcomes, as well as embryonic and placental growth and development. Mice and humans share physiological characteristics during both healthy pregnancy and those with adverse outcomes including immunological, hormonal, and fetal/placental development. While animal models created by genetic, surgical and pharmacological manipulation are highly valuable for studying the pathogenesis of IUGR, these models inadvertently introduce confounding factors when utilised for the assessment and development of new therapeutics. In contrast, the CBA/J × DBA/2J murine model^5^ spontaneously develops IUGR and embryonic/fetal mortality^6^,^7^ and shares features of the human IUGR complication, including inadequate maternal arterial remodelling at the implantation sites^8^,^9^ and utero-placental dysregulation with complement deposition on trophoblasts, increased inflammatory cell infiltration (neutrophil and monocyte) and tumour necrosis factor (TNF)-α expression in maternal decidua^6^,^10^,^11^.

Since identification of the abortion-prone phenotype in pregnant DBA/2J-mated CBA/J female mice in 1980, subsequent studies reported that this phenotype is exclusive to the American Jackson Laboratory-derived CBA/J female, with other CBA sub-strains (CBA/Ca, CBA/JHan, CBA/N) showing normal incidence^12^,^13^. This was believed to arise from semi-allogeneic rejection of the placenta by maternal immune cells due to differences in the murine major histocompatibility antigen, termed H-2 antigens which have accumulated by the separate breeding lines since 1935^13^,^14^; CBA/J (*H2k, Mlsd*) and CBA/Ca and CBA/N (*H2k, Mlsb*). However, ‘genetic drift’ may, in part, explain why more recent papers have identified new phenotypes in the CBA/J × DBA/2J model including fetal growth restriction^6^ and immunologically-mediated pre-eclampsia at mid gestation (15.5 dpc)^7^,^15^, though neither phenotype has been replicated in a term population (17.5–18.5 dpc)^16^.

As CBA/J mice are not currently available in Australia, and CBA/J and CBA/CaJ mice show differences in aging phenotypes^17^ we examined whether the Commonwealth-derived CBA/CaH.Arc mouse (Animal Resource Centre, Murdoch, Australia) can substitute for the American-derived CBA/J sub-strain in the spontaneous CBA × DBA/2J murine model. In this study we show that the CBA/CaH pregnant female develops an IUGR and embryonic/fetal mortality phenotype at mid-gestation (14.5 dpc) following allogeneic mating with DBA/2J males, suggesting that this sub-strain can be substituted where CBA/J mice are not available.

## Results

**Figure 1:**
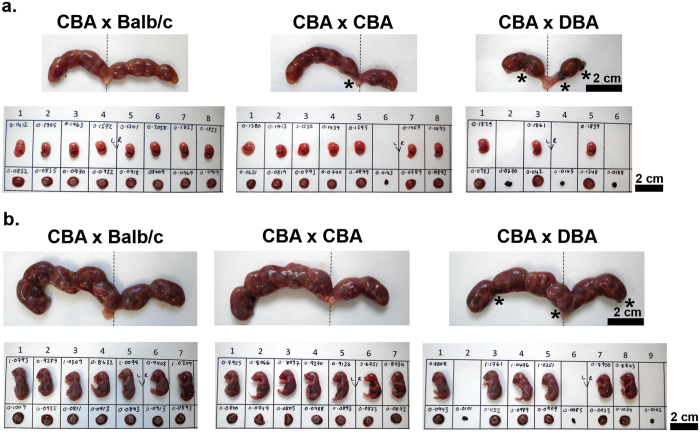
Macroscopic pregnancy outcomes of murine CBA/CaH × DBA/2J pregnancies. Representative photographs of gravid uteri harvested from CBA/CaH × Balb/c, CBA/CaH × CBA/CaH and CBA/CaH × DBA/2J pregnancies at (**a**) 14.5 dpc (N = 20 dams per strain) and (**b**) 18.5 dpc (N = 30 dams per strain). Fetuses and placentae were excised, measured, weighed and photographed. Weights of fetuses, placentae and resorptions are indicated in grams. Asteri denote resorption sites indicating embryonic/fetal mortality.

To establish whether CBA/CaH × DBA/2J pregnancies show the same propensity for embryonic/fetal mortality and IUGR as CBA/J females, 150 CBA/CaH females were placed with DBA/2J, Balb/c or CBA/CaH males overnight. Both mid-gestation (14.5 dpc, n = 20 matings per group) and term (18.5 dpc, n = 30 matings per group) time points were assessed to directly compare the fetal outcomes. Harvesting of gravid uteri, including the cervix, revealed the presence of resorption sites but showed no macroscopic uterine abnormalities in CBA/CaH × DBA/2J pregnancies at mid-gestation (Fig. 1a) or term (Fig. 1b).

### Embryonic/fetal mortality is increased in CBA/CaH × DBA/2J pregnancies

**Figure 2:**
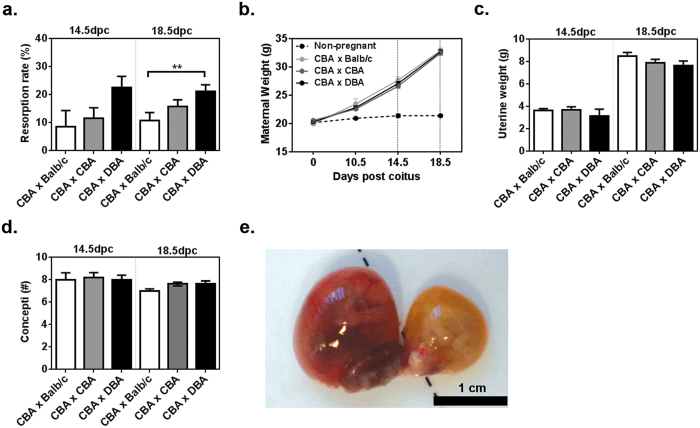
Maternal characteristics of the murine CBA/CaH × DBA/2J pregnancies. (**a)** Embryonic/fetal mortality (resorption) rate, (**b**) maternal body weight at mating, (**c**) uterine wet weight, and (**d**) number of concepti at 14.5 dpc (N = 20 dams per strain) and 18.5 dpc (N = 30 dams per strain). (**e**) Representative photograph of asymmetrical diamniotic/dichorionic (placentally fused) twins. All data are represented as mean ± SEM. **p < 0.01 vs value for CBA × Balb/c by One-way ANOVA with Tukey’s multiple comparison test.

The severest form of IUGR in our cohort is reflected as embryonic/fetal mortality (resorption). Overall, there was a significant increase in (CBA/CaH × DBA/2J) F1 embryonic/fetal mortality in when compared to (CBA/CaH × Balb/c) F1 at term (21.1 ± 2.4% vs 10.7 ± 2.9%, p = 0.0079; Fig. 2a), while mid-gestation did not reach significance (22.6 ± 3.89% vs 8.5 ± 5.89, p = 0.1569). Mortality in the (CBA/CaH × DBA/2J) F1 ranged widely from 0 to 63.4% at mid-gestation and between 0 to 55.5% in term populations, indicating that not all pregnancies are affected. This was particularly evident at term with 86.7% of CBA/CaH × DBA/2J litters containing resorptions, compared to 45.8% CBA/CaH × Balb/c and 79.2% for CBA/CaH × CBA/CaH pregnancies (Χ^2^ = 11.91, p = 0.0026). There was no statistical difference at mid-gestation.

Descriptors of the progression of resorption are ‘embryonic’, ‘bag of cells’ and ‘clot’ phases^18^, reflecting progressive loss of the fetal and placental structures via aseptic intravital autolysis. In our study evidence of all three stages was present, irrespective of the group analysed, with the clot phase most common in mid-gestation (70/72, 97.2%) and term (84/96, 87.5%) pregnancies. Embryonic phase resorptions were observed at term (6/96, 6.3%), but not mid-gestation, and were primarily associated with (CBA/CaH × Balb/c) F1 potentially indicating that fetal death and resorption takes place at a later time point in these pregnancies.

A range of factors have been found to influence embryonic/fetal mortality in mice and specifically the CBA/J × DBA/2J murine model. These include maternal nutrition, primigravidity, immunological environment, maternal age and overcrowding of one uterine horn^19^,^20^. However, irrespective of time-point we found no differences in maternal weight gain, uteri wet weight, or total number of concepti when compared to CBA/CaH × Balb/c (Fig. 2). The number of concepti in left *vs* right uterine horns of CBA/CaH × DBA/2J pregnancies was uneven (≥2 concepti difference between left and right horns; Χ^2^ = 7.370, p = 0.0251) when compared to CBA/CaH × Balb/c, but the ‘overcrowded’ horn was not associated with greater mortality when compared to the ‘uncrowded’ horn (Χ^2^ = 2.249, p = 0.3248; and shown Fig. 1).

While mortality rates did not differ, we considered whether CBA/CaH × DBA/2J pregnancies have abnormal spacing between implantation sites which may impact placental development leading to the increased mortality we observed. Using the incidence of diamniotic/dichorionic (placentally-fused) twins as a surrogate for abnormal implantation site, at term the incidence of twins was 3.6% (6/168) in (CBA/CaH × Balb/c) F1, 2.2% (4/183) in (CBA/CaH × CBA/CaH) F1, and 3.5% (8/230) in (CBA/CaH × DBA/2J) F1 and not statistically significant (Χ^2^ = 0.7434, p = 0.6895). Of the observed twin fetuses 50%, 25% and 25% respectively were asymmetrical (different size fetuses; example shown in Fig. 2e). At mid-gestation only one instance of diamniotic/dichorionic twins was observed in a CBA/CaH × DBA/2J pregnancy. These data suggest that overcrowding, with or without abnormal spacing, was not a significant contributing factor to the increased embryonic/fetal mortality observed in (CBA/CaH × DBA/2J) F1 and (CBA/CaH × CBA/CaH) F1.

### Proportion of IUGR fetuses is increased in CBA/CaH × DBA/2J pregnancies

**Figure 3:**
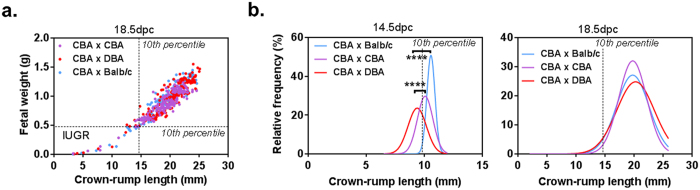
Fetal characteristics of the murine CBA/CaH × DBA/2J model. Fetuses were excised from gravid uteri at 14.5 and 18.5 dpc. (**a**) Crown-rump length showed a strong positive correlation with fetal wet weight. (**b**) Distribution of fetal crown-rump length for all strains where a length < 10^th^ percentile of (CBA × Balb/c) F1 were denoted as intrauterine growth restricted (IUGR; **b**). ***p = 0.005. ****p < 0.0001 vs (CBA × Balb/c) F1 by One-way ANOVA with Tukey’s multiple comparison test.

Studies using the CBA/J × DBA/2J murine model have reported that surviving fetuses show consistent and significant ‘IUGR’^6^, based on a statistical reduction in average fetal weight. To utilise a biologically relevant criterion for IUGR, we quantified the prevalence of IUGR in our murine pregnancies using the human clinical definition of <10^th^ percentile for gestational age, with fetuses from allogeneic (CBA/CaH × Balb/c) F1 as the population standard. Fetal wet weight and crown-rump length (CRL)^21^ were assessed and demonstrated a strong correlation at both time points (14.5 dpc r = 0.77 to 0.89; p = 0.0406 to p < 0.0001; and 18.5 dpc r = 0.91 to 0.94, p < 0.0001 Fig. 3a).

Using our definition, IUGR fetuses were denoted as those with a wet weight <0.1427g and <0.4725g and/or CRL <9.75 mm and <14.75 mm at 14.5 and 18.5 dpc, respectively. At mid-gestation, 47% of (CBA/CaH × DBA/2J) F1 fell below the 10^th^ percentile confirming a substantial IUGR phenotype at this time point (Fig. 3b). From 20 pregnant dams this equated to 46 IUGR fetuses, compared to 31 for (CBA/CaH × CBA/CaH) F1 and 13 for (CBA/CaH × Balb/c) F1. The number of IUGR fetuses dropped markedly at term with only 5–6 IUGR fetuses in all pregnancy groups. In contrast, the number of resorptions at term remained high; 46 in (CBA/CaH × DBA/2J) F1, 24 in (CBA/CaH × CBA/CaH) F1 and 14 in (CBA/CaH × Balb/c) F1. We propose that IUGR fetuses present at 14.5 dpc become non-viable by term (present as resorptions) resulting in a ‘perceived’ absence of IUGR at term in the CBA/CaH × DBA/2J pregnancies. There is precedence for this, with pregnant mice treated with 6 mg of the yeast extract Malucidin at 16–17 dpc, showing complete resorption of a fetus after 48 hr^22^. Even in large mammals, such as dogs, complete resorption of a fetus takes only 2–5 days and is preceded by reduced growth and/or development of the embryo and fetus^23^.

### Placentae from IUGR fetuses show histopathology

**Figure 4:**
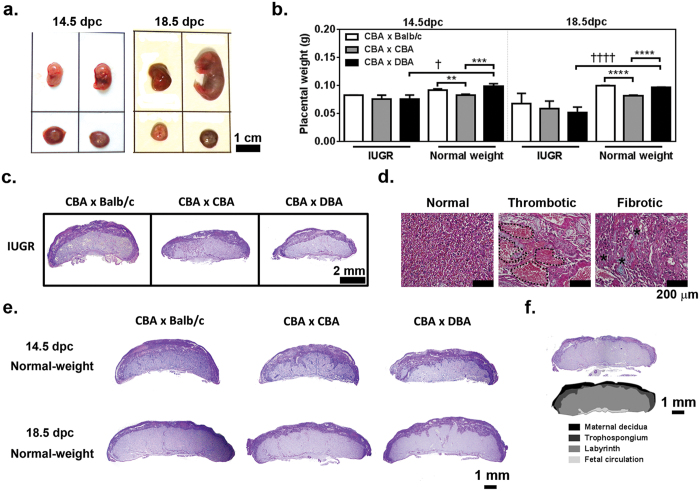
Macroscopic and histological characteristics of murine placentae from IUGR and normal-weight fetuses in the CBA/CaH × DBA/2J model. (**a**) Representative photograph of IUGR (<10^th^ %) and normal-weight (>10^th^ %) littermates at 14.5 dpc (left) and 18.5 dpc (right). (**b**) Placentae weight for IUGR and normal-weight fetuses represented as mean ± SEM. (**c**) PAS-stained sections of resorptions and placentae from IUGR fetuses (14.5 dpc) were assessed for differences in placental dimensions and the proportion devoted to the labyrinth, trophospongium and maternal decidua. (**d**) Comparison of normal labyrinthine tissue and areas of thrombotic (dotted line) and fibrotic tissue (asteri; presence of blue/green collagen) from IUGR associated placentae stained with fast-green Masson’s trichrome. (**e**) PAS-stained sections of placentae from normal-weight fetuses at 14.5 and 18.5 dpc, and (**f**) a (CBA/CaH × Balb/c) F1 fetuses at 18.5 dpc placentae that has been colour rendered to show the placental layers quantitated in Table 2. (**b**) **p < 0.01, ***p < 0.001 vs CBA × CBA by One-way ANOVA with Tukey’s multiple comparison test; ^†^p < 0.05 vs value for IUGR by unpaired two-tailed Student T test.

Macroscopically, the murine IUGR fetuses were developmentally delayed with smaller, thinner and/or pale placentae in comparison to normal-weight littermates (>10^th^ percentile; Fig. 4a). Quantitation of placental characteristics confirmed that IUGR placentae from (CBA/CaH × DBA/2J) F1 weighed on (mean) average 24% less at mid-gestation (p = 0.011) and 47% less at term (p < 0.0001) than their normal-weight littermates (Fig. 4b). Representative histological sections of IUGR placentae are shown in Fig. 4c (c.f. normal-weight in Fig. 4e). Evidence of pathological changes including thrombosis and fibrosis were present in placentae from IUGR fetuses of all groups (Fig. 4d), consistent with placental patho-histological observations of human IUGR placentae^24^,^25^.

Among the IUGR fetuses of all strains there were no differences in placental dimension at either time point (Table 1). In mice the primary site of feto-maternal exchange is the labyrinth, with the maternal decidua providing anchorage to the uterine wall and the fetal chorionic plate delivering blood to/from the umbilical cord. The proportion of IUGR placentae dedicated to the labyrinth layer was significantly less in (CBA/CaH × DBA/2J) F1 compared to (CBA/CaH × CBA/CaH) F1 and (CBA/CaH × Balb/c) F1 at mid-gestation, but did not reach significance at term (Table 1, and see Fig. 4f for colour rendering of placental layers). The trophospongium and maternal decidual layers were highly variable among the (CBA/CaH × DBA/2J) F1 cohort at 14.5 dpc indicating there may be a range of dysfunction among the placentae in this cohort (i.e. mild, moderate, severe).

One indicator of placental function, or the efficiency of placental nutrient transport, is the ratio of fetal wet weight to placental wet weight in grams^26^. In addition, with the placenta acting as a nutrient sensor and storage site for glucose (as glycogen) during pregnancy, we sought to determine whether there are changes in the proportion of glycogen-containing cells, and by extension the energy repository, available for fetal growth and development of (CBA/CaH × DBA/2J) F1. There was no significant difference in placental efficiency or the proportion of glycogen-containing cells at either time point between the (CBA/CaH × DBA/2J) F1 and (CBA/CaH × Balb/c) F1 IUGR placentae, although trends toward reduced efficiency and glycogen storage were present (Table 1). Meanwhile, the IUGR placentae of (CBA/CaH × CBA/CaH) F1 showed the highest placental efficiency of the three strains (Table 1).

### Comparison of placental morphology in IUGR and normal-weight fetuses

Normal-weight data is provided in Table 2 and micrographs in Fig. 4e. The p-values for the statistical comparison between placentae of IUGR and normal-weight fetuses of the same strain are provided in Supplementary Table I. Consistent with reduced placental weight, the IUGR placentae of all strains were significantly smaller in dimension (diameter, thickness and/or cross-sectional midline area) than their normal-weight littermates with the differences more pronounced at term than mid-gestation (Fig. 4a,e, and Supplementary Table I). When compared to normal-weight fetuses at mid-gestation, the placentae of IUGR fetuses from (CBA/CaH × DBA/2J) F1, but not the other strains, had smaller labyrinth (39.7 vs 52.4%, p < 0.0001) and larger trophospongium (41.4 vs 24.7%; p < 0.0001) (Supplementary Table I). Combined with the aforementioned analysis of placental structure of IUGR placentae, this may indicate that although each strain has IUGR fetuses, the histopathology of the (CBA/CaH × DBA/2J) F1 IUGR fetuses is more severe at this time point.

At term all strains demonstrated significantly greater placental efficiency for normal-weight fetuses compared to IUGR (all p < 0.0001; Table 1), though only the (CBA/CaH × Balb/c) F1 IUGR placentae showed differences in placental layer composition compared to normal-weight fetuses; less labyrinth (48.6 vs 63.5%; p* < 0.0001*), and larger trophospongium (29.1 vs 19.8%; p* < 0.0001),* maternal decidua (22.3 vs 16.7%; p = 0.0004) and glycogen storage (14.1 vs 9.5%; p* < 0.0001;* Table 1).

### Normal-weight fetuses of CBA/CaH × DBA/2J pregnancies show placental abnormalities

The increase in embryonic/fetal mortality, IUGR and abnormal placental structure in (CBA/CaH × DBA/2J) F1 IUGR placentae raised the question of whether there are also perturbations of the placenta evident for fetuses that macroscopically appear normal and have normal weight. In short, is there a predisposition in the (CBA/CaH × DBA/2J) F1 placenta that leads to IUGR and embryonic/fetal mortality? In the normal-weight fetal population, placentae from (CBA/CaH × DBA/2J) F1 were significantly smaller in diameter (p < 0.0001) and midline cross-sectional area (p = 0.0006) at term and significantly higher proportion of labyrinth and lower proportion of trophospongium at mid-gestation and term, when compared to (CBA/CaH × Balb/c) F1 (Fig. 4e and Table 2). Therein while these fetuses still achieved a normal body weight there are notable differences in the morphology of their placentae which may impact placental function.

Despite having reduced total placental area and greater proportion of labyrinthine layer (Table 2), which may have suggested altered surface area for feto-maternal exchange, there was no difference in placental efficiency between the placentae of (CBA/CaH × DBA/2J) F1 and normal-weight (CBA/CaH × Balb/c) F1 (Table 2). At mid-gestation, but not term, the placentae from (CBA/CaH × DBA/2J) F1 contained 55% more glycogen-containing cells compared to placentae from (CBA/CaH × CBA/CaH) F1 (p = 0.0009) and 27% more than (CBA/CaH × Balb/c) F1 (p = 0.09; Fig. 4e and Table 2). Placentae from (CBA/CaH × CBA/CaH) F1 had the lowest proportion of glycogen-containing cells at both 14.5 (8.19 ± 0.53%) and 18.5 dpc (8.60 ± 0.29%; Table 2), consistent with the lowest placental weights for normal-weight fetuses (Fig. 4b). Whether these differences in glycogen-storage are causal for reduced fetal growth or compensatory is unclear.

**Figure 5:**
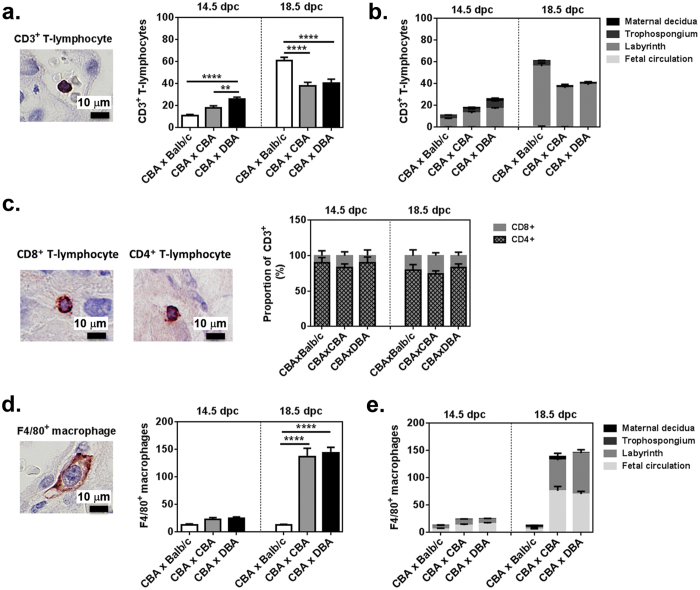
Inflammatory infiltrate of murine placentae from normal-weight fetuses at 14.5 and 18.5 dpc. (**a**) Representative micrograph and quantitation of CD3^+^T-lymphocytes in placentae and (**b**) their distribution among the placental layers. (**c**) The proportion of CD4^+^ helper T-lymphocytes and CD8^+^ CTLs to the placental T-lymphocyte infiltrate. (**d**) Representative micrograph and quantitation of F4/80^+^ macrophages and (**e**) their distribution among the placental layers. In (**a**–**c**) micrographs from placentae of (CBA/CaH × Balb/c) F1 fetuses at 18.5 dpc are shown to demonstrate positive staining of the immune cell types. Data are represented as mean ± SEM. N = 34–50 placentae/strain/dpc as outlined in Table 2. **p < 0.01, ****p < 0.0001, as indicated by One-way ANOVA with Tukey’s multiple comparison test.

A hallmark of placentae from IUGR pregnancies is marked inflammatory infiltrate^11^. While macrophages and T-lymphocytes are required for normal placentation, excessive and/or prolonged inflammation contributes to the pathology of IUGR by damaging the fetal-maternal interface, promoting trophoblast cell death^9^,^10^,^27^. At mid-gestation placentae from (CBA/CaH × DBA/2J) F1 normal-weight fetuses contained significantly more CD3^+^ T-lymphocytes than (CBA/CaH × CBA/CaH) F1 (1.4-fold, p < 0.01) or (CBA/CaH × Balb/c) F1placentae (2.6-fold, p < 0.0001; Fig. 5a). While the absolute number of CD3^+^ T-lymphocytes was higher in all groups at term, intriguingly there was 1.5-fold more CD3^+^ T-lymphocytes in (CBA/CaH × Balb/c) F1 placentae than (CBA/CaH × CBA/CaH) F1 and (CBA/CaH × DBA/2J) F1 placentae (both p < 0.0001). In all groups and time points >85% of CD3^+^ T-lymphocytes localised to the labyrinth (Fig. 5b). Subsequent analysis for CD4^+^ helper T-lymphocytes and CD8^+^ CTLs showed that while the absolute number of CD3^+^ T-lymphocytes differed among the groups, the proportion of CD4^+^ helper vs CD8^+^ CTLs was not significantly different; >83% CD4^+^ helper T-lymphocytes at mid gestation and >74% at term (Fig. 5c).

Two populations of F4/80^+^ macrophages are present in placenta; maternal haematopoietic-derived macrophages and fetal yolk sac-derived macrophages (Hofbauer cells)^28^. In addition to differences in origin, fetal macrophages predominantly localise to the chorionic villi (labyrinth in mice) and fetal circulation, and maternal macrophages to the maternal decidua^28^. (CBA/CaH × DBA/2J) F1 placentae had 1.8- fold more F4/80^+^ macrophages than (CBA/CaH × Balb/c) F1 placentae at mid-term and 11-fold more at term (Fig. 5d). These changes were also mimicked in (CBA/CaH × CBA/CaH) F1. Within the placentae, 30–50% of macrophages localised to labyrinth and 50–70% to the fetal circulation (chorionic plate; Fig. 5e). Our data shows that there is altered temporal and spatial distribution of T-lymphocyte and macrophages even in the placentae (CBA/CaH × DBA/2J) F1 fetuses that achieve a normal-weight.

## Discussion

The data in this study demonstrates that the IUGR and embryonic/fetal mortailty phenotype of the CBA/J × DBA/2J model can also be elicited by using the Commonwealth-derived CBA/CaH substrain. First, prevalence of IUGR and mortality was higher at 14.5 dpc in (CBA/CaH × DBA/2J) F1 than (CBA/CaH × Balb/c) F1. Second, in all strains IUGR fetuses were associated with lighter, smaller placentae with histopathology and reduced materno-fetal exchange as evidenced by smaller labyrinth and lower placental efficiencies when compared to normal-weight fetuses of the same strain. Finally, despite some fetuses obtaining ‘normal’ body weight, the placentae from these (CBA/CaH × DBA/2J) F1 show altered glycogen storage and considerable T-lymphocyte and macrophage inflammatory infiltration compared to (CBA/CaH × Balb/c) F1. These findings suggest that there is a range of placental pathology in the CBA/CaH × DBA/2J pregnancy which dictates the fetal outcome; severe pathology leads to early resorption, moderate to IUGR at 14.5 dpc with subsequent demise by term, and mild which supports live birth.

Among inbred murine strains there is a spectrum of gestational lengths, fetal and placental weights, maternal weight gain and number of concepti^29^. While gestational length and concepti are largely dependent upon maternal genotype^29^, which was unchanged in our study, fetal and placental weight are dependent on the genetic composition of the embryo. Of our three inbred strains, the syngeneic (CBA/CaH × CBA/CaH) F1 had the lowest fetal and placental weights of our normal-weight cohorts, consistent with the 14–20% lower fetal and placental weight reported in allogeneic *vs* syngeneic inbred matings^30^,^31^,^32^. The acquirement of heterosis in F1 fetuses is shown to precede implantation, with blastocysts of syngeneic pregnancies containing more cells than allogeneic pregnancies at the same gestational age^33^. As placentation continues the mixed genetic background of the fetuses may become more influential with differences in remodelling of the uterine vasculature and maternal immunological response associated with paternal MHC expression^34^. Yet, precisely how the mixed genetic background of (CBA/CaH × DBA/2J) F1 fetuses leads some to achieve normal-weight while others are growth restricted or die is unknown.

Technological advancements and greater appreciation of the placenta as an immunomodulatory organ have provided increasing evidence that dysregulation of placentation, and not a loss of maternal immune tolerance against the semi-allogeneic fetus *per se,* underlie human and murine (CBA/J × DB/2JA) IUGR and mortality^8^,^9^,^10^,^35^. Following implantation fetal trophoblasts invade the maternal decidua and partner resident natural killer (NK) cells^36^ to remodel spiral arteries by inducing apoptosis of endothelial cells and vascular smooth muscle cells by soluble Fas ligand^37^,^38^,^39^ and tumour necrosis factor-alpha-related apoptosis-inducing ligand. Apoptotic cells are phagocytosed, typically by decidual macrophages, though first trimester trophoblasts are phagocytic^40^ and may also play a role in clearing these cells during spiral artery remodelling. Such aberrations in the function of the trophoblasts or NK cells results in a failure to convert the maternal spiral arteries into low resistance, high capacity vessels^39^. Subsequent perfusion of the placenta with maternal blood at high pressure incites trophoblast damage via shear stress and mechanical damage^41^ leading to thrombotic and fibrotic events as noted in the IUGR placentae of all murine strains in our study, and infarcts, loss of tertiary villi and increased syncytial knots (associated with syncytiotrophoblast apoptosis) among the placentae of human IUGR newborns^42^. Such injury to the human placental villi or murine labyrinth reduces the surface area of materno-fetal exchange contributing to IUGR.

Delineation of the initial mechanism that renders trophoblasts or decidual NK cells less effective at spiral artery remodelling is a relatively new field of research and is complicated by access to very early human placental tissue and the differences between invasiveness of murine and human trophoblasts. From the CBA/J × DBA/2J model there is evidence of inadequate placentation via poor maternal arterial remodelling at the implantation sites^8^ and perturbations of genomic DNA methylation in the maternal decidua^43^. Other studies report inflammatory mechanisms, with complement deposition on trophoblasts with increased neutrophil and monocyte infiltration and TNF-α expression in maternal decidua and reduced trophoblast giant cells in placentae of resorbing fetuses^6^. Both abnormal DNA methylation and complement deposition appear to distinguish normally developing fetuses from those which resorb, even within the same litter^6^,^43^. Our study compliments this evidence wherein perturbations in placental size and morphology were observed in placentae of IUGR fetuses when compared to normal-weight littermates. In humans, these parameters dictate the number of maternal spiral arteries that can be remodelled and subsequently influences the surface area of nutrient exchange in the placenta^44^ affecting fetal growth and development.

It is understandable that the outcome of any fetus is dependent on the severity of impact to the placenta, as the key regulator of both oxygen and nutrients. Mild alterations in placental morphology or inflammation below a threshold do not observably impact on weight or CRL but may evoke epigenetic or anatomical changes that alter ongoing fetal-adult development. To our knowledge the CBA × DBA model has not been examined as far as pup development following birth, largely due to the need to excise uteri and fetuses for assessment of embryonic/fetal mortality and IUGR. However, as ultrasound biomicroscopy technology becomes more cost-effective, such non-invasive assessments of viable *vs* non-viable murine fetuses on the basis of heart beat^45^, as well as *in situ* murine fetal growth measurements^46^ will be reported and enable long term outcomes to be assessed in this model. Of particular note would be cognitive function and behavioural impacts as these are commonly associated with children who were IUGR^47^, despite ‘catching up’ in weight after birth. This will be essential for assessment of novel therapeutics to reduce embryonic/fetal mortality and IUGR using the CBA × DBA/2J model.

In summary, we have provided a comprehensive assessment of fetal outcome and placental dimension and immune infiltrate temporal and spatial anomalies at two gestational time points (14.5 and 18.5 dpc), with both allogeneic and syngeneic control matings, to demonstrate that the CBA/CaH × DBA/J is an equivalent murine model of IUGR and embryonic/fetal mortality to the CBA/J × DBA/2J model for assessment of pregnancy pathology and/or novel therapeutics.

## Methods

### Animals and Tissue Preparation

All experiments were approved and performed in accordance with the guidelines of the Northern Sydney Local Health District Animal Research Ethics Committee, Australia (ACEC 1311-015A). Twelve-week old virgin female CBA/CaH.Arc and male CBA/CaH.Arc (*H2k*), Balb/c.Arc (*H2d*), and DBA/2J.Arc (*H2d*) mice were purchased from the Animal Resources Centre (Murdoch, Australia). Animals were provided food and water *ad libitum* and kept under specific pathogen-free conditions with controlled humidity and light conditions.

Timed syngeneic CBA/CaH × CBA/CaH, and allogeneic CBA/CaH × Balb/c or CBA/CaH × DBA/2J matings were performed by pairing females with males overnight. Detection of the vaginal plug denoted 0.5 days post coitum (dpc). At 14.5 dpc (mid-gestation; N = 20 dams per strain) and 18.5 dpc (term; N = 30 dams per strain) pregnant mice were sacrificed by cervical dislocation and the gravid uteri, including the cervix, were harvested and weighed. Subsequently, each fetus and placentae were dissected, weighed, the fetal crown-rump length measured, and the litter photographed. The number of concepti and embryonic/fetal mortality (resorption) rate were also recorded. Embryonic/fetal mortality was defined as including ‘macerated pale fetuses (embryonic)’, bag of cells, and ‘clot’^18^. Pink/perfused fetuses were considered viable. Overall there was ≥80% pregnancy success in each group. In the term population, two pregnant dams from each group delivered pre-term at 17.5 dpc and were excluded from subsequent analyses.

### Histology and Immunohistochemistry

#### Paraffin-embedded tissues

Four μm sections of paraffin-embedded resorptions and placentae were cut sagittal to the midline and stained with haemotoxylin and eosin (general morphology), periodic acid Schiff’s (PAS) procedure (glycogen-positive cells in the trophospongium), or fast-green Masson’s trichrome stain (fibrosis). Immunohistochemistry was performed for T-lymphocytes (CD3e; 1 μg/ml DAKO) and monocyte/macrophages (F4/80; 2 μg/ml Santa Cruz) following proteinase K (2 mg/ml) antigen retrieval. Normal rat and rabbit IgG served as isotype controls (DAKO).

#### Frozen tissues

Resorptions and placentae snap-frozen in liquid nitrogen were embedded in Optimal Cutting Temperature compound. Eight μm cryosections were immunohistochemically stained for helper T-lymphocytes (CD4; 0.5 μg/ml Santa Cruz) and cytotoxic T-lymphocytes, CTL (CD8; 0.5 μg/ml Santa Cruz). Normal rat IgG served as isotype control (DAKO).

### Placental Structure Quantification

Quantitation of the placental dimensions (diameter, thickness, and midline cross-sectional area) were analysed using haemotoxylin and eosin stained sections and the placental layers (labyrinth, trophospongium and maternal decidua) using PAS-stained sections. All histological analyses were performed using two parallel sections per IUGR and normal-weight placentae taken at least 100 μm apart. Low magnification micrographs were taken using a Nikon Eclipse C*i* microscope fitted with a DS-Fi1 digital camera and DS-U2 control unit and 2x Plan Achromat objective lenses. From the scale bar placental diameter, thickness, and midline cross-sectional area were measured using ImageJ 1.48v software (http://imagej.nih.gov/ij) and expressed in millimetres. The area attributed to the labyrinth, trophospongium and maternal decidual layers was assessed and expressed as a percentage of the total area of the placenta (excluding fetal circulation structures). The percentage of glycogen-containing trophoblasts in PAS-stained sections was quantified by magenta-coloured pixels in micrographs using ImageJ 1.48v software (http://imagej.nih.gov/ij). A macro to automate the analysis was modified from the freely available ImageJ resource at http://imagej.net/docs/examples/stained-sections/index.html (NIH) and utilised the green channel and a magenta colour threshold of (0, max/1.4). For each section the data was calculated as the area of magenta coloured pixels/placental midline cross-sectional area*100 and expressed as glycogen-containing cells as a proportion (%) of the placental area.

### Statistical Analyses

Normality of the data for each outcome was determined by the D’Agostino & Pearson omnibus K2 normality test (GraphPad Prism; NIH), except the IUGR 18.5 dpc cohorts due to low sample size (normal distribution was assumed using the Central Limit Theorem as the placentae are derived from normally distributed populations; see Fig. 3b). Data are expressed as mean ± standard error of the mean (SEM). Comparison between groups at 14.5 dpc and 18.5 dpc was performed using unpaired two-tailed Student t-test and between groups of a single time point by One-way ANOVA with Tukey’s multiple comparison test. Association between crown-rump length and fetal weight was determined by Pearson correlation. The Chi square test was used to compare the number of concepti between left *vs* right uterine horns, the frequency of perinatal mortality in ‘crowded’ *vs* ‘uncrowded’ horns, incidence of placentally–fused twins, and differences in proportion of litters with resorbing fetuses *vs* those without. A p-value < 0.05 was considered statistically significant for all analyses.

## Additional Information

**How to cite this article**: McKelvey, K. J. *et al*. Embryonic/fetal mortality and intrauterine growth restriction is not exclusive to the CBA/J sub-strain in the CBA × DBA model. *Sci. Rep.* **6**, 35138; doi: 10.1038/srep35138 (2016).

## Supplementary Material

Supplementary Information

## Figures and Tables

**Table 1:** Characteristics of placentae from IUGR fetuses.

	**14.5 dpc**			**ANOVA**
	**CBA × Balb/c**	**CBA × CBA**	**CBA × DBA**	***p-value***
Placentae (N)	13	31	46	
Diameter (mm)	8.30 ± 0.34	7.06 ± 0.29	6.99 ± 0.94	*0.8478*
Thickness (mm)	2.28 ± 0.11	2.21 ± 0.317	3.66 ± 0.04	*0.1177*
Area (mm^2^)	19.37 ± 1.03	14.32 ± 1.94	21.52 ± 3.97	*0.1541*
Labyrinth (%)	51.34 ± 2.54^a^	62.03 ± 2.67^af^	39.69 ± 2.28	*0.0006*
Trophospongium (%)	25.64 ± 2.36	18.09 ± 1.38^a^	41.39 ± 8.51	*0.0229*
Maternal decidua (%)	23.01 ± 1.37	19.88 ± 1.28	18.91 ± 10.74	*0.4961*
Placental efficiency (fetal/placental weight)	1.72 ± 0.02	1.87 ± 0.16^ae^	1.20 ± 0.17	*0.0084*
Glycogen-containing cells (% placenta)	10.84 ± 0.0	15.86 ± 1.19^c^	9.85 ± 1.24	*0.0022*
	**18.5 dpc**			**ANOVA**
Placentae (N)	5	6	6	
Diameter (mm)	7.63 ± 0.28	7.36 ± 0.55	6.83 ± 0.59	*0.1883*
Thickness (mm)	2.42 ± 0.18	2.33 ± 0.06	2.07 ± 0.20	*0.6188*
Area (mm^2^)	18.71 ± 2.33	15.64 ± 1.47	12.83 ± 1.55	*0.1240*
Labyrinth (%)	48.64 ± 2.99	64.25 ± 3.25	60.81 ± 5.11	*0.0529*
Trophospongium (%)	29.08 ± 4.37	19.45 ± 1.72	24.16 ± 2.39	*0.0691*
Maternal decidua (%)	22.29 ± 2.39	16.31 ± 2.85	18.07 ± 5.19	*0.2443*
Placental efficiency (fetal/placental weight)	1.21 ± 0.27	1.82 ± 0.53	0.60 ± 0.37	*0.1761*
Glycogen-containing cells (% placenta)	14.14 ± 2.25	8.74 ± 0.79^d^	9.78 ± 1.14	*0.0319*

Data are expressed as mean ±* SEM.*^*a*^p < 0.05, ^b^p < 0.01, ^c^p < 0.001 vs (CBA/CaH × DBA/2J) F1; ^d^p < 0.05, ^e^p < 0.01, ^f^p < 0.001 vs (CBA/CaH × Balb/c) F1 by One-way ANOVA with Tukey’s multiple comparison test. For each placentae two parallel sections taken 100 μm apart were analysed.

**Table 2:** Characteristics of placentae from normal-weight fetuses.

	**14.5 dpc**			**ANOVA**
	**CBA × Balb/c**	**CBA × CBA**	**CBA × DBA**	***p-value***
Placentae (N)	34	35	36	
Diameter (mm)	8.84 ± 0.35	8.85 ± 0.14	9.17 ± 0.14	*0.2810*
Thickness (mm)	2.67 ± 0.07	2.46 ± 0.04^a^	2.63 ± 0.05	*0.0250*
Area (mm^2^)	23.97 ± 1.01	22.35 ± 0.60	23.92 ± 0.69	*0.1809*
Labyrinth (%)	48.10 ± 1.09^a^	54.83 ± 0.96^af^	52.44 ± 0.80^d^	*0.0006*
Trophospongium (%)	28.61 ± 0.61^a^	23.46 ± 0.71^f^	24.75 ± 0.71^d^	*0.0012*
Maternal decidua (%)	23.29 ± 0.71	21.71 ± 0.83	22.81 ± 0.74	*0.4442*
Placental efficiency (fetal/placental weight)	1.86 ± 0.03	2.25 ± 0.14^e^	2.447 ± 0.36	*0.0068*
Glycogen-containing cells (% placenta)	10.00 ± 0.72	8.19 ± 0.53^c^	12.68 ± 0.98	*0.0004*
	**18.5 dpc**			**ANOVA**
Placentae (N)	50	44	46	
Diameter (mm)	10.38 ± 0.093^c^	9.69 ± 0.09^f^	9.45 ± 0.15^f^	*<0.0001*
Thickness (mm)	2.72 ± 0.03	2.50 ± 0.04^e^	2.65 ± 0.04	*0.0063*
Area (mm^2^)	26.05 ± 0.40^c^	21.58 ± 0.50^f^	23.22 ± 0.56^f^	<*0.0001*
Labyrinth (%)	63.51 ± 0.64^a^	48.64 ± 2.99^af^	65.78 ± 0.97^d^	<*0.0001*
Trophospongium (%)	19.79 ± 0.49	16.96 ± 0.52^e^	18.82 ± 0.59	*0.0088*
Maternal decidua (%)	16.66 ± 0.38	14.01 ± 0.55^e^	15.53 ± 0.68	*0.0094*
Placental efficiency (fetal/placental weight)	10.00 ± 0.24	11.20 ± 0.25^e^	10.41 ± 0.26	*0.0055*
Glycogen-containing cells (% placenta)	9.47 ± 0.16	8.60 ± 0.30^e^	9.23 ± 0.38	*0.0077*

Data are expressed as mean ± SEM. ^a^p < 0.05, ^b^p < 0.01, ^c^p < 0.001 vs (CBA/CaH × DBA/2J) F1; ^d^p < 0.05, ^e^p < 0.01, ^f^p < 0.001 vs (CBA/CaH × Balb/c) F1 by One-way ANOVA with Tukey’s multiple comparison test. For each placentae two parallel sections taken 100 μm apart were analysed.

## References

[b1] WHO. Expert Committee Report: Physical status: the use and interpretation of anthropometry. (World Health Organization, 1995). 8594834

[b2] Baschat A. Fetal responses to placental insufficiency: an update. Br. J. Obstet. Gynaecol. 111, 1031–1041 (2004). 10.1111/j.1471-0528.2004.00273.x 15383103

[b3] Alexander G. . US birth weight/gestational age-specific neonatal mortality: 1995–1997 rates for whites, hispanics, and blacks. Pediatrics. 111, e61–e66 (2003). 12509596 10.1542/peds.111.1.e61 PMC1382183

[b4] Ott W. An update on the ultrasonic diagnosis and evaluation of intrauterine growth restriction. Ultrasound Rev. Obstet. Gynecol. 5, 111–124 (2005).

[b5] Clark D. A., McDermott M. R. & Szewczuk M. R. Impairment of host-versus-graft reaction in pregnant mice: II. Selective suppression of cytotoxic T-cell generation correlates with soluble suppressor activity and with successful allogeneic pregnancy. Cell. Immunol. 52, 106–118 (1980). 6446406

[b6] Girardi G., Yarilin D., Thurman J., Holers V. & Salmon J. E. Complement activation induces dysregulation of angiogenic factors and causes fetal rejection and growth restriction. J. Exp. Med. 203, 2165–2175 (2006). 16923853 10.1084/jem.20061022 PMC2118387

[b7] Ahmed A., Singh J., Khan Y., Seshan S. & Girardi G. A new mouse model to explore therapies for preeclampsia. PLoS One. 5, e13663 (2010). 21048973 10.1371/journal.pone.0013663 PMC2965104

[b8] Dixon M. E., Chien E. K., Osol G., Callas P. W. & Bonney E. A. Failure of decidual arteriolar remodeling in the CBA/J × DBA/2 murine model of recurrent pregnancy loss is linked to increased expression of tissue inhibitor of metalloproteinase 2 (TIMP-2). Am. J. Obstet. Gynecol. 194, 113–119 (2006). 16389019 10.1016/j.ajog.2005.06.063

[b9] Whitley G. S. J. & Cartwright J. E. Trophoblast-mediated spiral artery remodelling: a role for apoptosis. J. Anat. 215, 21–26 (2009). 19215319 10.1111/j.1469-7580.2008.01039.x PMC2714635

[b10] Scifres C. M. & Nelson D. M. Intrauterine growth restriction, human placental development and trophoblast cell death. J. Physiol. 587, 3453–3458 (2009). 19451203 10.1113/jphysiol.2009.173252 PMC2742274

[b11] Mestan K. . Placental inflammatory response is associated with poor neonatal growth: preterm birth cohort study. Pediatrics. 125, e891–e898 (2010). 20308216 10.1542/peds.2009-0313

[b12] Chaouat G. The immunology of the fetus. 254–259 (CRC Press, 1990).

[b13] Bobe P. & Kiger N. Immunogenetic studies of spontaneous abortion in mice. III. Non-H-2 antigens and gestation. J. Immunogenet. 16, 223–231 (1989). 2614072 10.1111/j.1744-313x.1989.tb00465.x

[b14] Click R. & Adelmann A. Multi-gene/allele control of Mlsb of CBA/H. Immunogenetics. 29, 155–160 (1989). 2522413 10.1007/BF00373640

[b15] Qing X. . Targeted inhibition of complement activation prevents features of preeclampsia in mice. Kidney Int. 79, 331–339 (2001). 10.1038/ki.2010.393 20944547

[b16] Poudel R. . Effects of 2-methoxyestradiol administration in mouse models purported to show signs of preeclampsia and fetal growth restriction. Int. J. Reprod. Fertility & Sex. Health (2014).

[b17] Ohlemiller K. K., Dahl A. R. & Gagnon P. M. Divergent Aging Characteristics in CBA/J and CBA/CaJ Mouse Cochleae. Journal of the Association for Research in Otolaryngology. 11, 605–623 (2010). 20706857 10.1007/s10162-010-0228-1 PMC2975886

[b18] Milliam Stanley M. & Solderwall A. Morphological changes accompanying fetal resorption in the Golden Hamster. Am. J. Anat. 114, 539–549 (1964). 14167175 10.1002/aja.1001140311

[b19] Chavez D. J., McIntyre J. A., Colliver J. A. & Faulk W. P. Allogeneic matings and immunization have different effects on nulliparous and multiparous mice. J. Immunol. 139, 85–88 (1987). 3584989

[b20] Ho H. . Age, environment, and lymphocyte immunization influence the spontaneous resorption rate in the CBA/J × DBA/2J mouse model. Am. J. Reprod. Immunol. 31, 47–51 (1994). 8166947 10.1111/j.1600-0897.1994.tb00846.x

[b21] Rocha F. & Zalud I. In Donald School Textbook of Ultrasound in Obstetrics and Gynecology (eds Kurjak A. & Chervenak F. A.) Ch. 14, 213 (Jaypee Brothers Medical Publishers (P) Ltd, 2011).

[b22] Levi M., Manahan J. & Mandi I. Specific resorption of the mouse fetus. Obstet. Gynecol. 33, 11–19 (1969). 5813103 10.1097/00006250-196901000-00002

[b23] England G. C. W. & Russo M. Ultrasonographic characteristics of early pregnancy failure in bitches. Theriogenology. 66, 1694–1698 (2006). 16554089 10.1016/j.theriogenology.2006.01.028

[b24] Kotgirwar S., Ambiye M., Athavale S., Gupta V. & Trivedi S. Study of gross and histological features of placenta in intrauterine growth retardation. J. Anat. Soc. India. 60, 37–40 (2011).

[b25] Biswas S. Placental changes in idiopathic intrauterine growth restriction. OA Anatomy. 1, 11 (2013).

[b26] Wilson M. & Ford S. Comparative aspects of placental efficiency. Reprod. Suppl. 58, 223–232 (2001). 11980192

[b27] Cotechini T. . Inflammation in rat pregnancy inhibits spiral artery remodeling leading to fetal growth restriction and features of preeclampsia. J. Exp. Med. 211, 165–179 (2014). 24395887 10.1084/jem.20130295 PMC3892976

[b28] Pinhal-Enfield G., Vasan N. & Leibovich S. In Embryology - Updates and Highlights on Classic Topics (ed. Pereira L. V.) Ch. 6, 127–142 (InTech, 2012).

[b29] Murray S. A. . Mouse gestation length is genetically determined. PLoS One. 5, e12418 (2010). 20811634 10.1371/journal.pone.0012418 PMC2928290

[b30] McCarthy J. C. Genetic and environmental control of foetal and placental growth in the mouse. Animal Science. 7, 347–361 (1965).

[b31] McLaren A. Genetic and environmental effecrs on foetal and placental growth. J. Reprod. Fertil. 9, 79–98 (1965). 14257721 10.1530/jrf.0.0090079

[b32] Hetherington C. M. The decidual cell reaction, placental weight, foetal weight and placental morphology in the mouse. J. Reprod. Fertil. 25, 417–424 (1971). 5579694 10.1530/jrf.0.0250417

[b33] Gates A., Doyle L. & Noyes R. A physiological basis for heterosis in hybrid mouse fetuses. Amer. Zool. 1, 449 (1961).

[b34] Madeja Z. . Paternal MHC expression on mouse trophoblast affects uterine vascularization and fetal growth. Proceedings of the National Academy of Sciences of the United States of America. 108, 4012–4017 (2011). 21300875 10.1073/pnas.1005342108 PMC3053985

[b35] Chaouat G. . An insight into normal and pathological pregnancies using large-scale microarrays: lessons from microarrays. J. Reprod. Immunol. 89, 163–172 (2011). 21329986 10.1016/j.jri.2010.12.006

[b36] Wallace A. E., Fraser R. & Cartwright J. E. Extravillous trophoblast and decidual natural killer cells: a remodelling partnership. Hum. Reprod. Update. 18, 458–471 (2012). 22523109 10.1093/humupd/dms015 PMC3373213

[b37] Ashton S. V. . Uterine spiral artery remodeling involves endothelial apoptosis induced by extravillous trophoblasts through Fas/FasL interactions. Arterioscler. Thromb. Vasc. Biol. 25, 102–108 (2005). 15499040 10.1161/01.ATV.0000148547.70187.89 PMC4228192

[b38] Harris L. K. . Invasive trophoblasts stimulate vascular smooth muscle cell apoptosis by a fas ligand-dependent mechanism. Am. J. Pathol. 169, 1863–1874 (2006). 17071607 10.2353/ajpath.2006.060265 PMC1780207

[b39] Fraser R. . Impaired decidual natural killer cell regulation of vascular remodelling in early human pregnancies with high uterine artery resistance. J. Pathol. 228, 322–332 (2012). 22653829 10.1002/path.4057 PMC3499663

[b40] Choy M. Y. & Manyonda I. T. The phagocytic activity of human first trimester extravillous trophoblast. Human reproduction (Oxford, England). 13, 2941–2949 (1998). 10.1093/humrep/13.10.2941 9804259

[b41] Burton G. J., Woods A. W., Jauniaux E. & Kingdom J. C. Rheological and physiological consequences of conversion of the maternal spiral arteries for uteroplacental blood flow during human pregnancy. Placenta. 30, 473–482 (2009). 19375795 10.1016/j.placenta.2009.02.009 PMC2697319

[b42] Veerbeek J. H. . Placental pathology in early intrauterine growth restriction associated with maternal hypertension. Placenta. 35, 696–701 (2014). 25052232 10.1016/j.placenta.2014.06.375

[b43] Brown L. Y., Bonney E. A., Raj R. S., Nielsen B. & Brown S. Generalized Disturbance of DNA Methylation in the Uterine Decidua in the CBA/J × DBA/2 Mouse Model of Pregnancy Failure. Biol. Reprod. 89, 120 (2013). 24108302 10.1095/biolreprod.113.113142 PMC4434988

[b44] Salafia C., Charles A. & Maas E. Placenta and fetal growth restriction. Clin. Obstet. Gynaec. 49, 236–256 (2006). 10.1097/00003081-200606000-00007 16721104

[b45] Flores L. E., Hildebrandt T. B., Kühl A. A. & Drews B. Early detection and staging of spontaneous embryo resorption by ultrasound biomicroscopy in murine pregnancy. Reprod. Biol. Endocrinol. 12, 38–38 (2014). 24886361 10.1186/1477-7827-12-38 PMC4037759

[b46] Mu J., Slevin J. C., Qu D., McCormick S. & Adamson S. L. *In vivo* quantification of embryonic and placental growth during gestation in mice using micro-ultrasound. Reprod. Biol. Endocrinol. 6, 34 (2008). 18700008 10.1186/1477-7827-6-34 PMC2527569

[b47] Geva R., Eshel R., Leitner Y., Valevski A. & Harel S. Neuropsychological outcome of children with intrauterine growth restriction: A 9-year prospective study. Pediatrics. 118, 91–100 (2006). 16818553 10.1542/peds.2005-2343

