# Supplementary material for: Embryonic/fetal mortality and intrauterine growth restriction is not exclusive to the CBA/J sub-strain in the CBA × DBA model
Source: Sci Rep. 2016 Oct 21;6:35138. doi: 10.1038/srep35138 (PMC5073309; doi:10.1038/srep35138)
Supplement: Supplementary Information [file srep35138-s1.pdf]

Embryonic/fetal mortality and intrauterine growth restriction is not exclusive to the CBA/J sub-strain in the CBA x DBA model

Kelly J McKelvey, Vanessa M Yenson, Anthony W Ashton, Jonathan M Morris, Sharon A McCracken

Supplementary Table I. P-values for comparison of IUGR vs normal-weight fetuses by placental parameter and gestation

	**14.5 dpc**			**18.5dpc**		
	**CBAx Balb/c**	**CBAx CBA**	**CBAx DBA**	**CBAx Balb/c**	**CBAx CBA**	**CBAx DBA**
Diameter (mm)	*0.3563*	*0.0048*	*0.0004*	*<0.0001*	*<0.0001*	*<0.0001*
Thickness (mm)	*0.0087*	*0.2098*	*<0.0001*	*0.0278*	*0.0403*	*0.0003*
Area (mm^2)	*0.0113*	*0.0034*	*0.3633*	*<0.0001*	*<0.0001*	*<0.0001*
Labyrinth (%)	*0.1848*	*0.0859*	*<0.0001*	*<0.0001*	*0.0221*	*0.1287*
Trophospongium (%)	*0.1269*	*0.0853*	*<0.0001*	*<0.0001*	*0.0694*	*0.0055*
Maternal decidua (%)	*0.8415*	*0.6040*	*0.2948*	*0.0004*	*0.2316*	*0.3337*
Placental efficiency (fetal/placental weight)	*0.3117*	*0.3120*	*0.0527*	*<0.0001*	*<0.0001*	*<0.0001*
Glycogen-containing cells (% placenta)	*0.5360*	*0.0006*	*0.4561*	*<0.0001*	*0.8358*	*0.6357*

Statistical analyses of IUGR vs normal-weight fetal data of the same strain was performed using unpaired two-tailed Student-T tests. Only p-values are provided here, corresponding N, mean ± SEM data can be found in Tables I and II.
